# Supplementary figures and images for: The oxidative fumarase FumC is a key contributor for E. coli fitness under iron-limitation and during UTI
Source: PLoS Pathog. 2020 Feb 27;16(2):e1008382. doi: 10.1371/journal.ppat.1008382 (PMC7064253; doi:10.1371/journal.ppat.1008382)

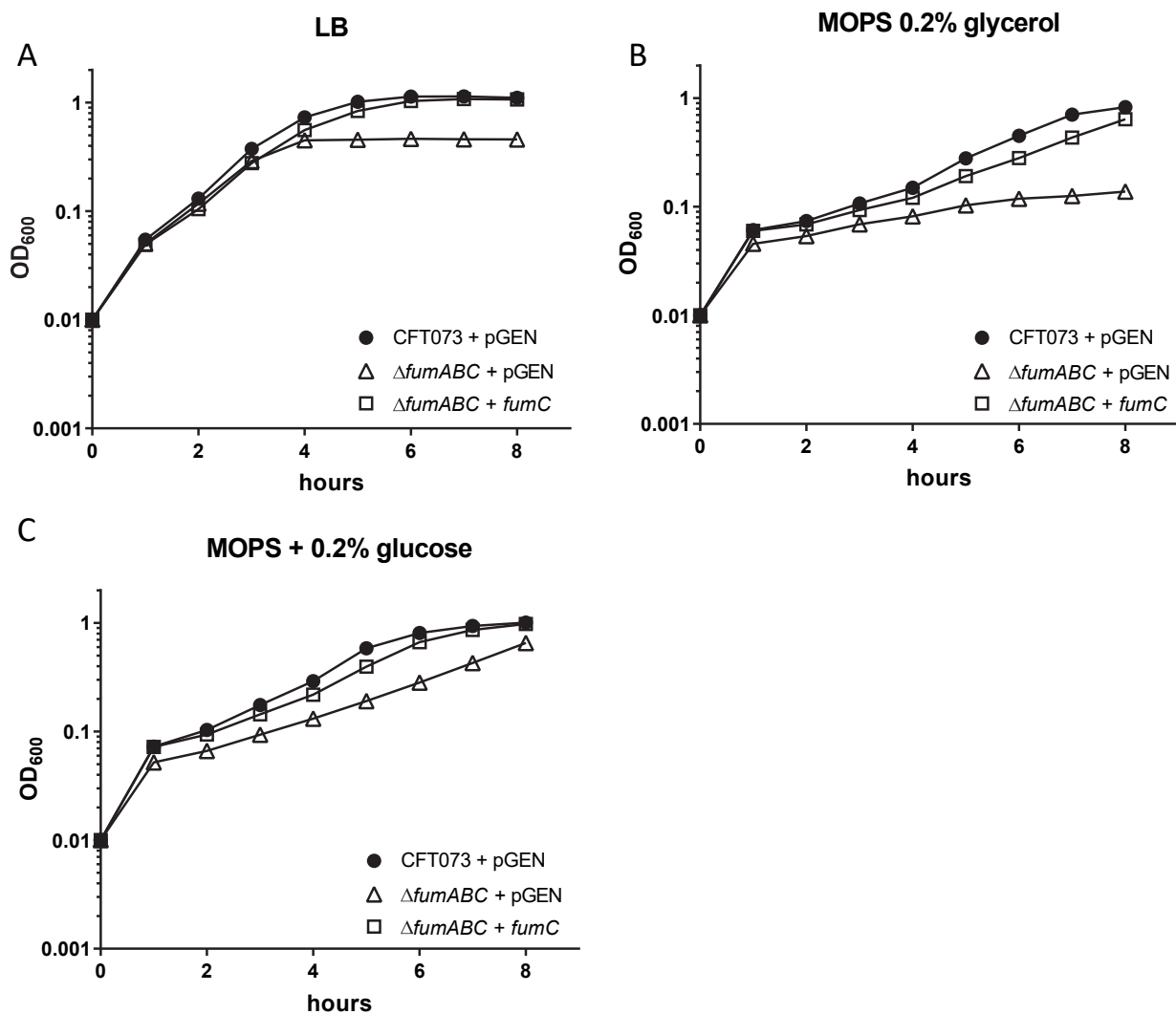

Fig S1

Supplement: S1 Fig — The growth defect of triple mutant strain fumABC was complemented with pGEN-fumC following growth in (A) LB medium, (B) defined medium containing 0.2% glycerol, and (C) defined medium containing 0.2% glucose. OD600 values were recorded each hour and the mean of three independent trails is plotted. (PDF) [file ppat.1008382.s001.pdf]

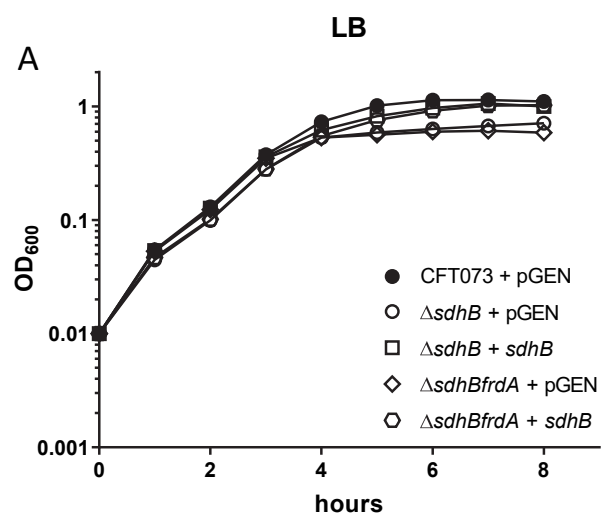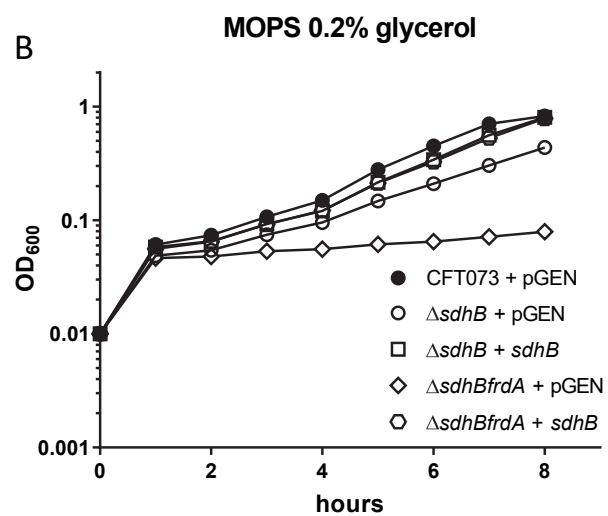

Fig S2

Supplement: S2 Fig — The growth defect of single mutant strain sdhB and double mutant strain sdhBfrdA was complemented with pGEN-sdhB following growth in (A) LB medium and (B) defined medium containing 0.2% glycerol. OD600 values were recorded each hour and the mean of three independent trails is plotted. (PDF) [file ppat.1008382.s002.pdf]

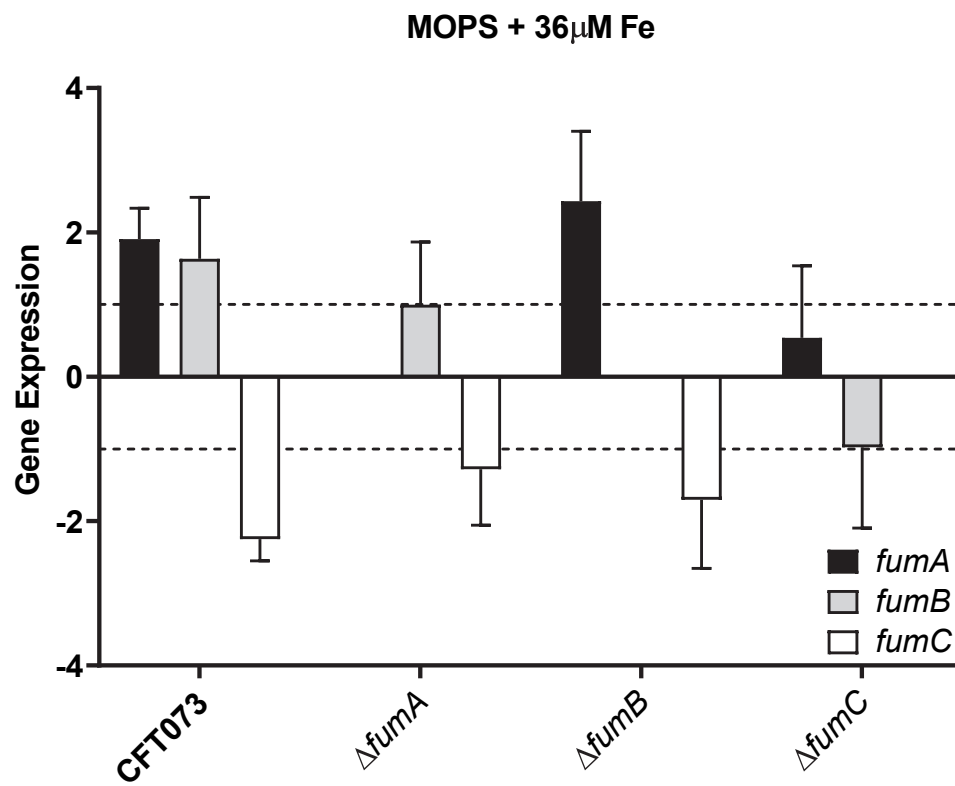

Fig S3

Supplement: S3 Fig — (A) Wild-type CFT073 and single fumarase mutant strains were grown shaking in defined medium containing 0.2% glucose with and without 36 μM FeCl3 at 37°C until OD600 = 0.5 for RNA isolation. qPCR was performed to examine gene expression of fumarase genes, fumA, fumB, and fumC. The comparative threshold cycle (CT) method was used to determine the relative log2 fold-change of each strain in iron-replete medium compared to iron-limitation. (PDF) [file ppat.1008382.s003.pdf]

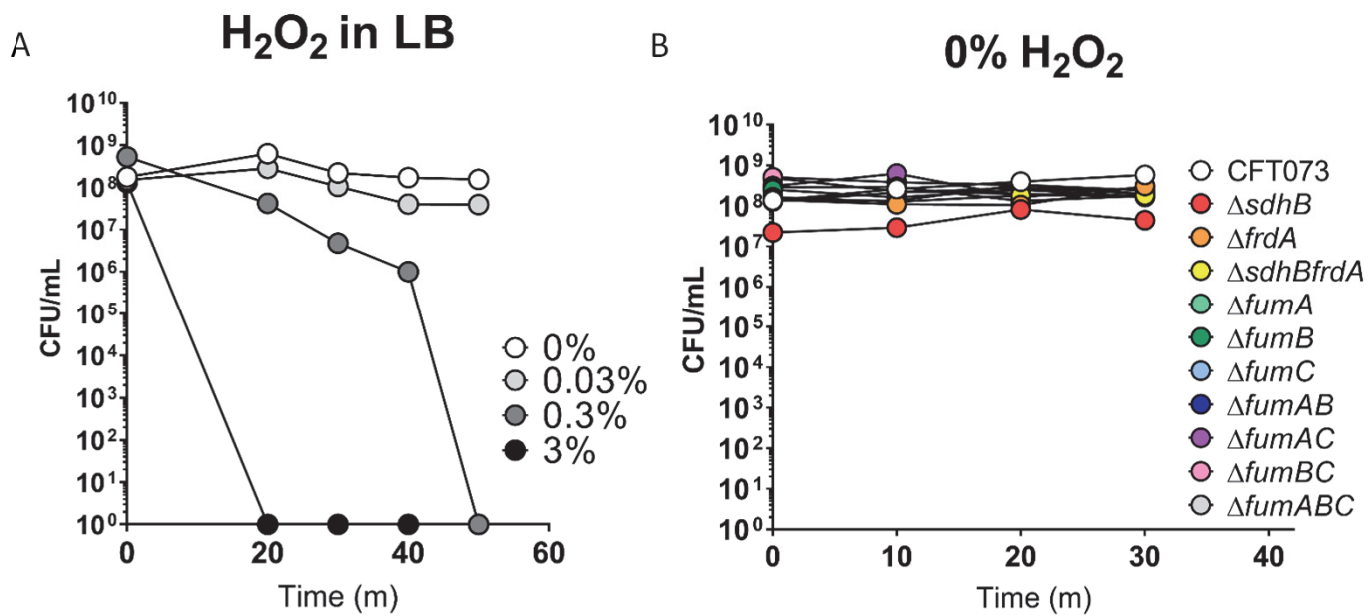

Fig S4

Supplement: S4 Fig — (A) Wild-type CFT073 was examined in LB medium containing either 0%, 0.03%, 0.3%, or 3% H2O2 to determine its susceptibility resulting in at least three logs of killing. Samples were collected every 10 minutes, diluted, and plated on LB agar to determine CFU. (B) Wild-type CFT073 and the oxidative and reductive TCA pathway mutant strains were examined in LB medium containing 0.0% H2O2. Samples were collected every 10 minutes, diluted, and plated on LB agar to determine CFU. (PDF) [file ppat.1008382.s004.pdf]

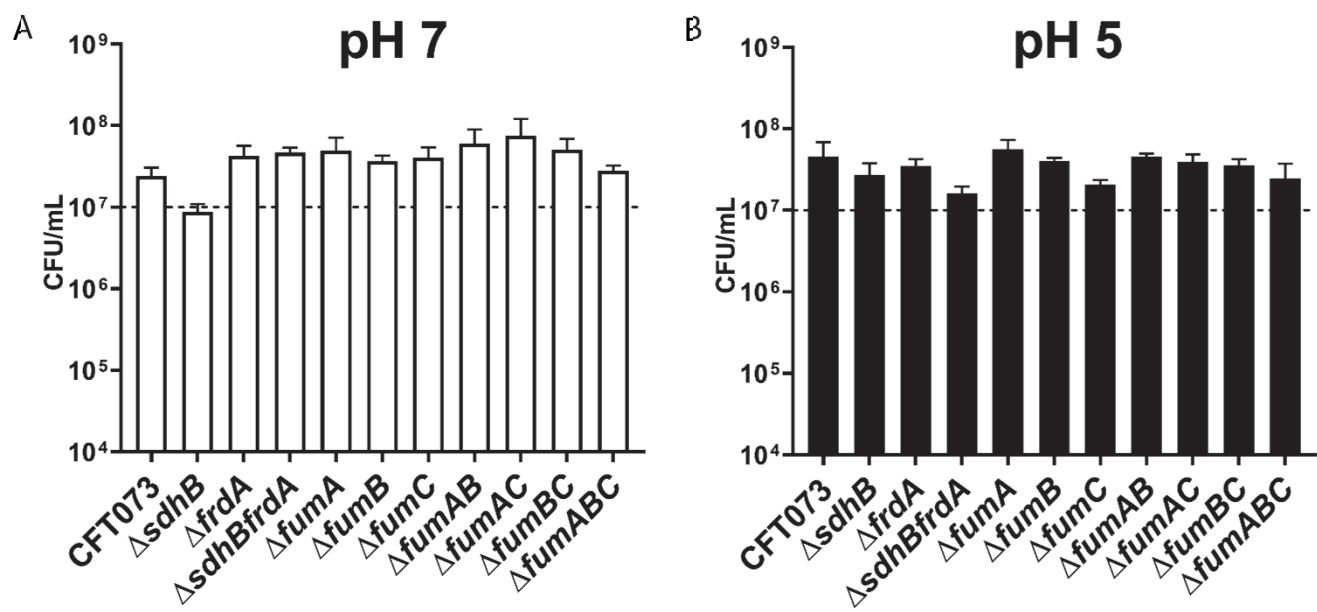

Fig S5

Supplement: S5 Fig — Wild-type CFT073 and the oxidative and reductive TCA pathway mutant strains were inoculated at 107 CFU/ml and incubated in (A) 100 mM MES buffered LB, pH 7.0, and (B) 100 mM MES buffered LB, pH 5.0, for 60 minutes, diluted, and plated on LB agar to determine CFU. The dashed line represents the intended input CFU/ml at time zero. Bars display the mean of three independent trials, error bars show SEM values. (PDF) [file ppat.1008382.s005.pdf]

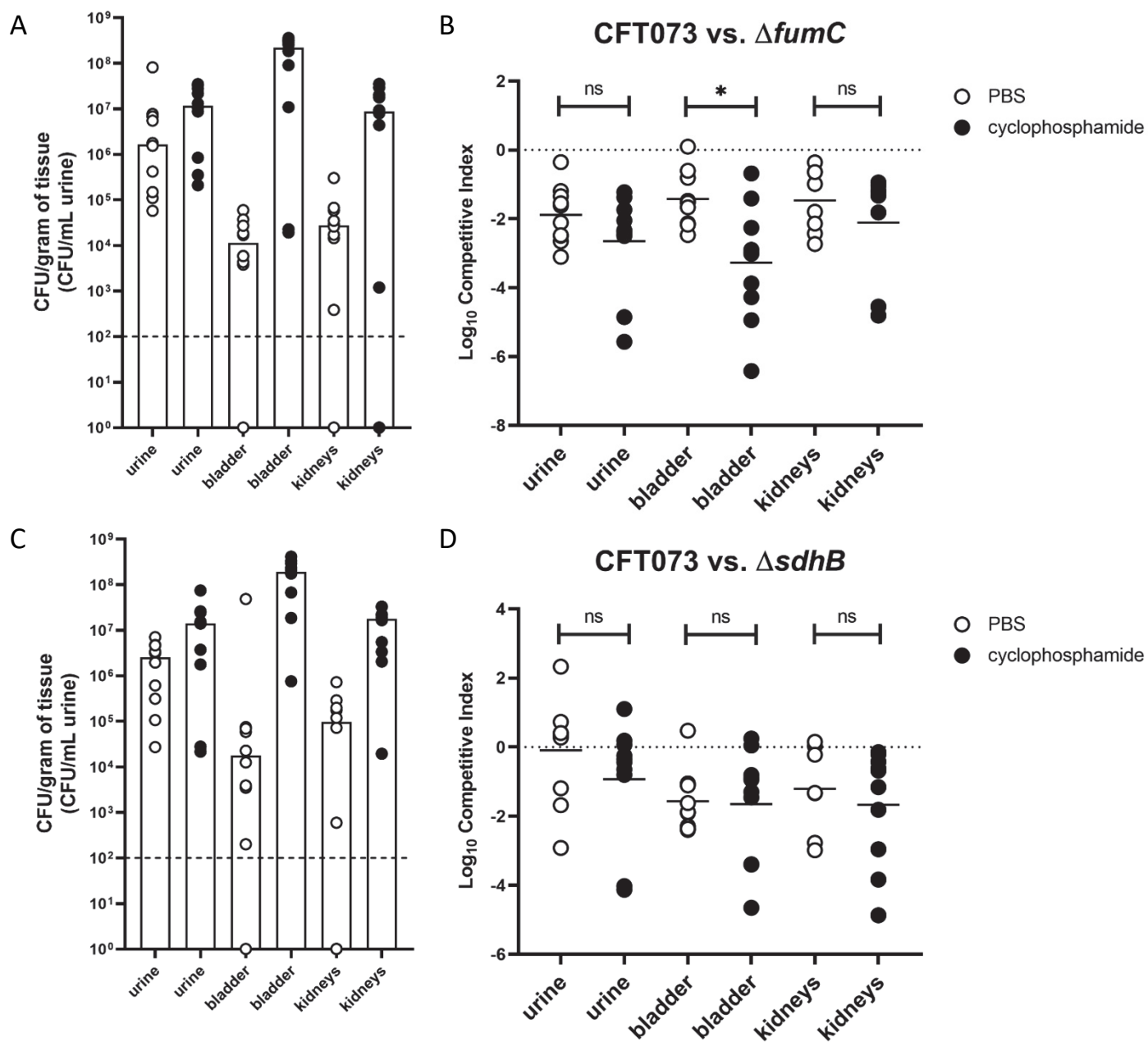

Fig S6

Supplement: S6 Fig — Mice were treated with PBS control (open symbols) or cyclophosphamide (closed symbols) at 96 h and 24 h prior to infection. Blood samples were obtained from both groups for analysis. Wild-type CFT073 and either the (A, B) fumC mutant strain or (C, D) sdhB mutant strain were mixed in a 1:1 ratio and transurethrally infected into CBA/J mice. Urine, bladder, and kidneys were harvested 48 h post-infection and CFU/ml urine or g tissue was measured (A, C). Log10 CI was calculated for each individual mouse organ (B, D). Bars display the mean. CI>1 indicates a fitness advantage and CI<1 indicates a fitness defect of the mutant. Significant differences in colonization (*P<0.05) were determined using the Mann Whitney test. (PDF) [file ppat.1008382.s006.pdf]

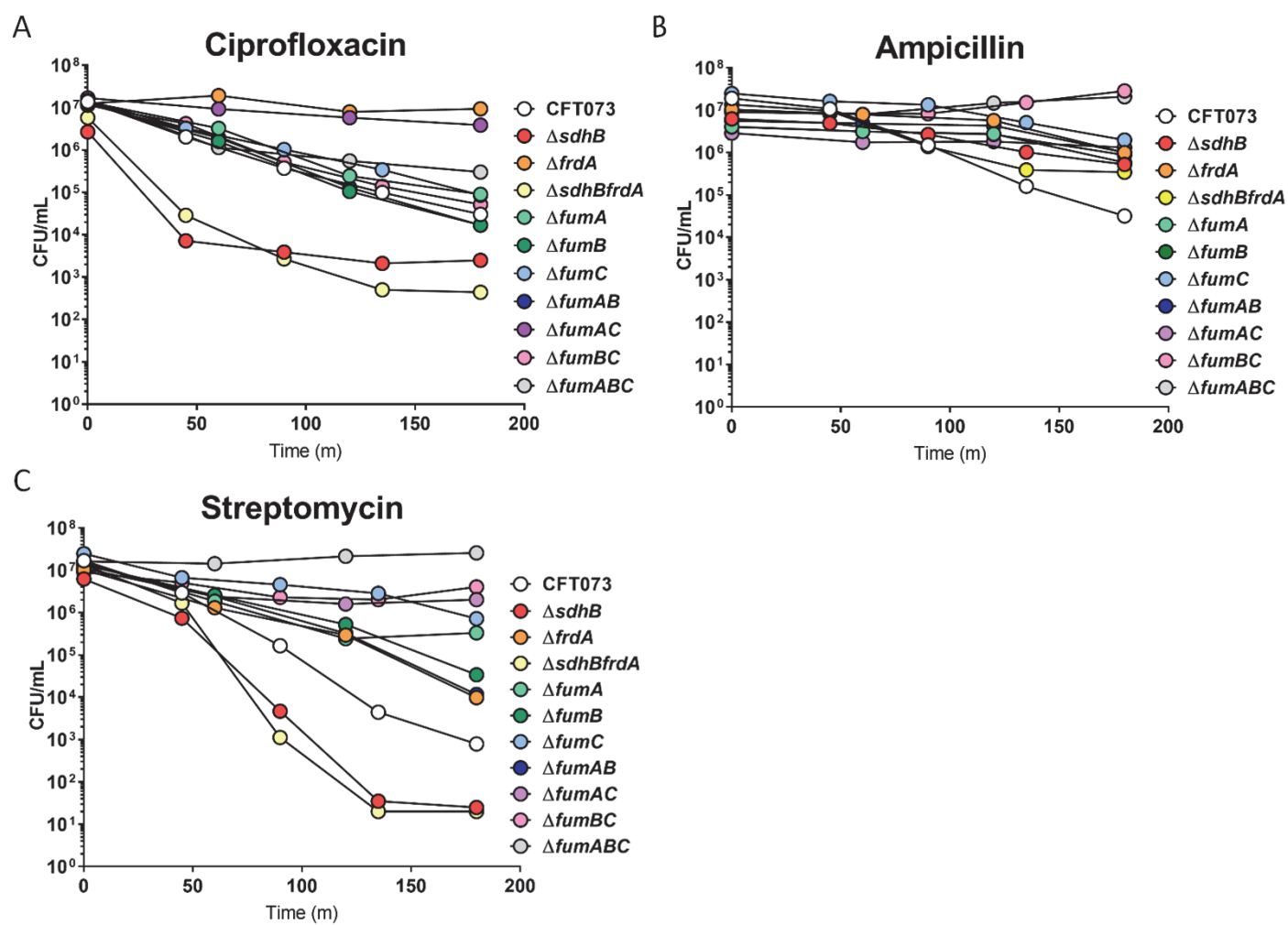

Fig S7

Supplement: S7 Fig — Wild-type CFT073 and the oxidative and reductive TCA pathway mutant strains were examined in LB medium containing (A) ciprofloxacin 250 ng/ml (B) ampicillin 5 μg/ml or (C) streptomycin 5 μg/ml. Samples were collected over the course of 180 minutes, diluted and plated on LB agar to determine CFU. Each symbol represents the average CFU of three independent trials with each indicated strain. (PDF) [file ppat.1008382.s007.pdf]

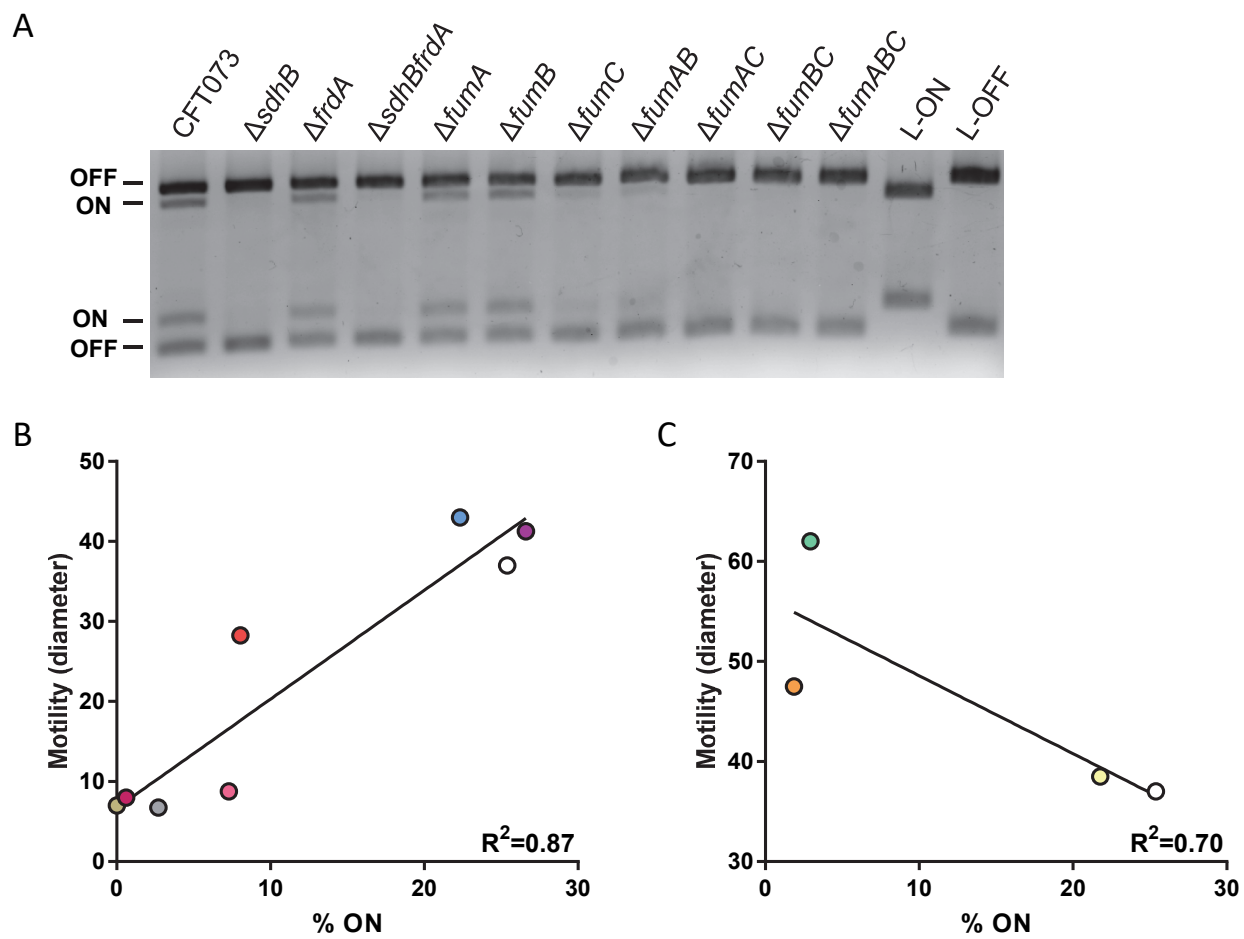

Figure S9

Supplement: S9 Fig — (A) For the invertible element (IE) assay, strains were grown statically in LB at 37°C for 18 h and standardized to OD600 = 0.5 prior to PCR amplification, subsequent digestion with SnaBI and running on a 3% agarose-1× TAE (Tris-acetate-EDTA buffer) gel. The size of PCR products representing on or off orientation of type 1 fimbriae (fim) promoter are indicated. (B) The relationship between motility and fim phase position was determined by plotting the motility diameter against the fim L-ON percentage of the population for each fumarase mutant and wild-type CFT073. Linear regression determined R2 = 0.87. (C) The relationship between motility and fim phase position was determined by plotting the motility diameter against the fim L-ON percentage of the population for the succinate dehydrogenase and fumarate reductase mutants with wild-type CFT073. Linear regression determined R2 = 0.70. (PDF) [file ppat.1008382.s009.pdf]

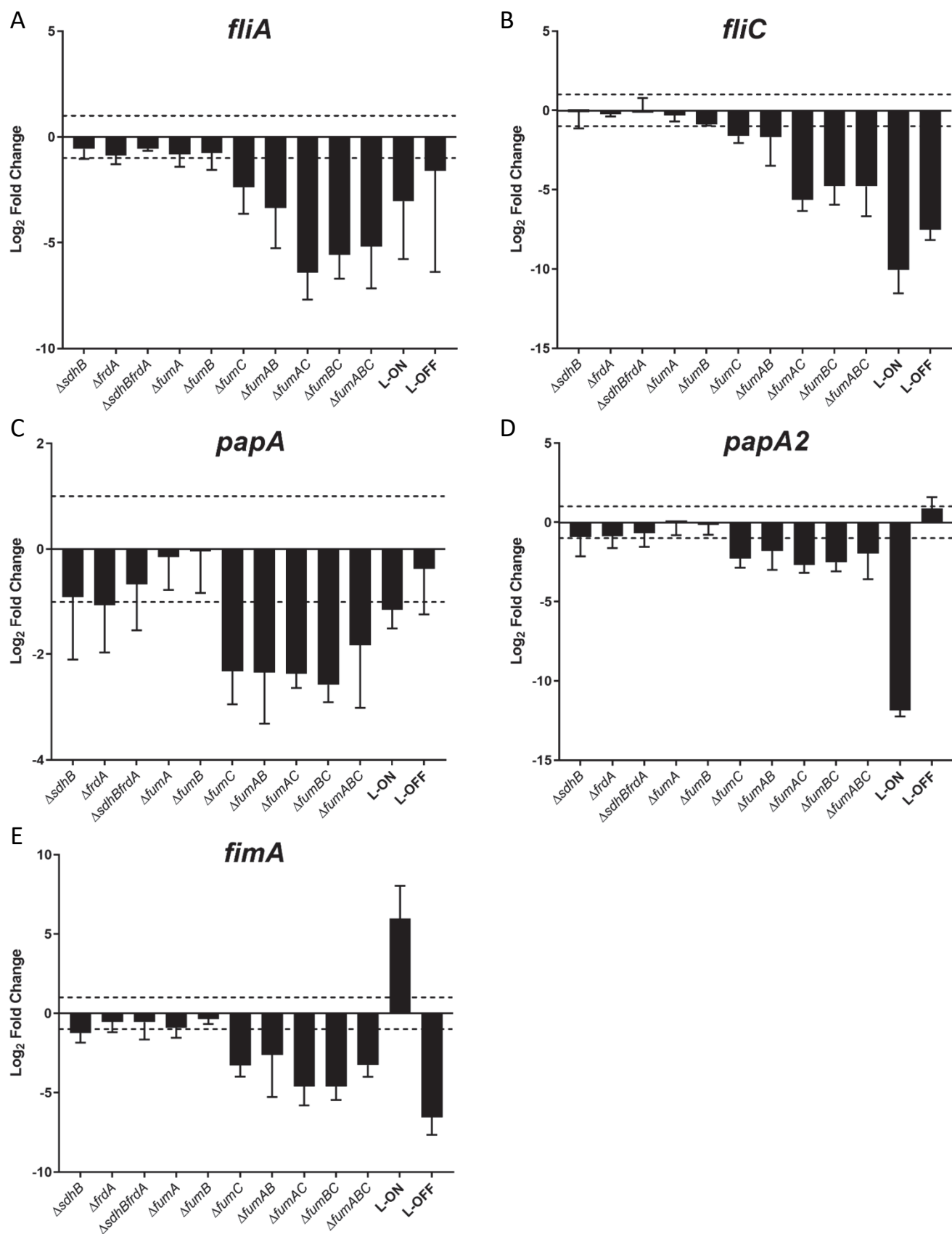

Figure S10

Supplement: S10 Fig — (A-E) Strains were grown in LB shaking at 37°C until OD600 = 0.5 for RNA isolation. qPCR was performed to examine gene expression of flagellar genes (fliA and fliC) and fimbrial genes (fimA, papA, and papA2). The comparative threshold cycle (CT) method was used to determine the relative log2 fold-change compared to wild-type CFT073. Bars denote the mean of three independent RNA isolation events, error bars are SEM. (PDF) [file ppat.1008382.s010.pdf]
