# Supplementary material for: The oxidative fumarase FumC is a key contributor for E. coli fitness under iron-limitation and during UTI
Source: PLoS Pathog. 2020 Feb 27;16(2):e1008382. doi: 10.1371/journal.ppat.1008382 (PMC7064253; doi:10.1371/journal.ppat.1008382)
Supplement: S8 Fig — The Minimum Bacterial Concentration (MBC) of (A) ciprofloxacin 0.002–32 μg/ml (B) ampicillin 0.016–256 μg/ml or (C) streptomycin 0.064–24 μg/ml observed for wild-type CFT073 and the oxidative and reductive TCA cycle mutant strains were recorded following overnight incubation on Mueller-Hinton agar with antibiotic test strips in anaerobic chambers. The values shown are averages from two independent trials. (PDF) [file ppat.1008382.s008.pdf]

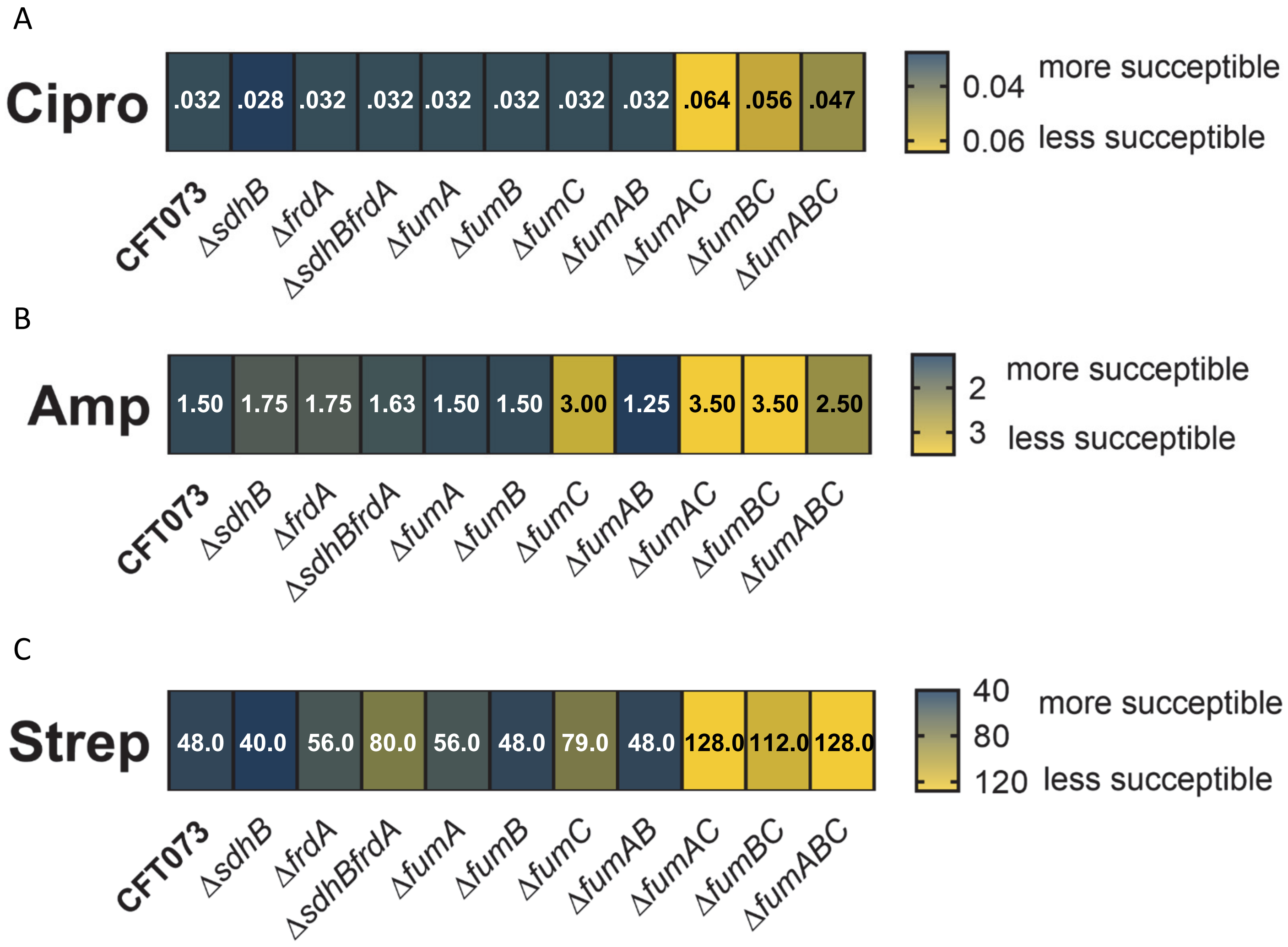
A
Cipro
.032
.028
.032
.032
.032
.032
.032
.032
.064
.056
.047
0.04
0.06
more succeptible
less succeptible
CFT073
ΔsdhB
ΔfrdA
ΔsdhBfrdA
ΔfumA
ΔfumB
ΔfumC
ΔfumAB
ΔfumAC
ΔfumBC
ΔfumABC
B
Amp
1.50
1.75
1.75
1.63
1.50
1.50
3.00
1.25
3.50
3.50
2.50
2
3
more succeptible
less succeptible
CFT073
ΔsdhB
ΔfrdA
ΔsdhBfrdA
ΔfumA
ΔfumB
ΔfumC
ΔfumAB
ΔfumAC
ΔfumBC
ΔfumABC
C
Strep
48.0
40.0
56.0
80.0
56.0
48.0
79.0
48.0
128.0
112.0
128.0
40
80
120
more succeptible
less succeptible
CFT073
ΔsdhB
ΔfrdA
ΔsdhBfrdA
ΔfumA
ΔfumB
ΔfumC
ΔfumAB
ΔfumAC
ΔfumBC
ΔfumABC

Fig S8
