# Supplementary material for: The oxidative fumarase FumC is a key contributor for E. coli fitness under iron-limitation and during UTI
Source: PLoS Pathog. 2020 Feb 27;16(2):e1008382. doi: 10.1371/journal.ppat.1008382 (PMC7064253; doi:10.1371/journal.ppat.1008382)
Supplement: Attachment — Submitted filename: Respiration_responses to reviewers.docx [file ppat.1008382.s011.docx]

*Reviewer's Responses to Questions*

***Part I - Summary*** *Please use this section to discuss strengths/weaknesses of study, novelty/significance, general execution and scholarship.

Reviewer #1: In this study, Himpsl, Shea, et al. investigate the energy and central carbon metabolism of UPEC in the context of urinary tract colonization. Previous work had suggested that mutants unable to perform an oxidative TCA cycle are less fit in bladder colonization model, while mutants unable to perform a branched TCA cycle did not. Here, the authors provide an exhaustive and in-depth characterization of mutants defective in the conversion of malate to fumarate as an extension of this work. In their mouse model or urinary tract infection, lack of FumC activity was associated with a decrease in bladder and kidney colonization. They also report that the fitness defect generated in the absence of an oxidative TCA cycle can be compensated by mutations in the fumarate reductase. In vitro, susceptibility of mutants to exogenous stress (acid stress, peroxide stress) and motility defects correlate with the observed defects in the mouse model. These data suggest that defects in the TCA cycle lead to increased susceptibility to ROS and other antimicrobials produced from alterations in the activity and composition of the respiratory chain.

While we have a good understanding of the E. coli metabolism of under standard laboratory conditions, the metabolism associated with host colonization by pathogenic E. coli is understudied. As such, the topic of this manuscript should be of great interest to the field. The careful and comprehensive analysis of mutant phenotypes in vitro and in vivo is commendable –certainly a lot of hard work. These findings should be helpful for the interpretation of previous findings on the function of the central carbon metabolism in E. coli and other Enterobacteriaceae. At this point, I my only concerns are regarding the writing and the novelty of the overall conclusions, as outlined below.*

**We appreciate the encouraging words and hope to have addressed your concerns with new experiments and with a thorough editorial overhaul.**  *Reviewer #2: This manuscript by Himpsl et al builds on two previous publications by the Mobley group that implicated enzymes within the TCA cycle as determinants of UPEC fitness during UTI. Specifically, the previous studies showed that the fumarate dehydrogenase FumC and the succinate dehydrogenase SdhB are important for UPEC fitness within the urinary tract, where deletion of the fumarate reductase FrdA increased fitness. The current study digs deeper into this system, showing that deletion of frdA counters the fitness defect observed when sdhB is mutated. Results are also presented showing that only fumC, among the three fumarate dehydrogenases encoded by UPEC, is important for UPEC fitness during UTI. A series of in vitro experiments suggest that the main defects associated with deletion of sdhB and fumC are due to increased oxidative stress, and perhaps altered membrane integrity. The data are robust and, for the most part, presented in a way so that even non-experts in metabolism can appreciate the findings. The work increases our understanding of UPEC fitness determinants, though a lack of clear mechanisms for the many phenotypes described and some overlap with previous studies may be viewed as problematic.*
**We appreciate the thoughtful critique and have made a sincere effort to address these shortcomings, not least of which is the overlap with previous studies. We also took strides to better understand the mechanisms underlying some of the observed phenotypes with admittedly varying degrees of success.**

***Part II – Major Issues: Key Experiments Required for Acceptance*** *Please use this section to detail the key new experiments or modifications of existing experiments that should be absolutely required to validate study conclusions.

Generally, there should be no more than 3 such required experiments or major modifications for a "Major Revision" recommendation. If more than 3 experiments are necessary to validate the study conclusions, then you are encouraged to recommend "Reject".


Reviewer #1: 1. The writing is fairly dense to a point that it is hard to read and understand what the authors are trying to say. For example, in the abstract, the authors use gene symbols, enzyme names, and abbreviations. This is all technically correct and I understand what the authors want to convey, yet I fear that my trainees and readers outside the field, this is not easily accessible. The findings are really interesting, but this might be lost on most readers.*

**This is very helpful. It would be unfortunate to dissuade potential readers with a poorly written abstract. The abstract has now been revised to be more accessible to a wider audience. Additionally, the discussion has been shortened substantially to eliminate redundancy and to make our main findings more clear.**

*2. While the study provides a good framework for the interpretation of the in vivo phenotypes, it is difficult to understand what the authors have really found – what is the conceptual advance? It comes as no surprise that manipulation of the central metabolism will result in pleiotropic effects and that the field should be careful in designing experiments.*

**This is a very good point. We have made an effort to clarify what we believe we have really found. In fact, it was both of the reviewer’s comments that helped us to better understand our own findings through additional experiments and thoughtful reflection of all of our data. We believe the conceptual advance is now more clearly presented; that modular components in central metabolism are important for UPEC during UTI. Importantly, the title of the revised manuscript has also been revised to reflect this clarity. One key finding is that while FumA is typically the preferred fumarase, the environmental conditions, iron-limitation in the urinary tract, promotes a shift to favor FumC reactions. This is why we believe loss of FumC has an outsized impact on UPEC fitness.**

*3. The in vitro experiments suggest a few testable hypotheses as to why certain mutants are attenuated in vivo. It would be important to take this correlation between in vitro phenotyping and in vivo disease modeling a step further and experimentally test these ideas. For example, the authors surmise that neutrophil-derived products might be a stressor of UPEC. One would predict that neutrophil depletion as well as pharmacological or genetic manipulation would rescue the observed E. coli mutant phenotypes.*

**We could have anticipated this point much better, so we apologize for that. The reviewer is quite correct. We have now performed co-challenge experiments with *sdhB* and *fumC* mutant strains in mice that have been pharmacologically manipulated to be neutropenic. While not the result we initially expected, these data were quite helpful to rule out one of our favorite hypotheses and served to greatly reduce our speculative commentary that pervaded the earlier version of this study. Although we are not yet willing to die on this hill, the blood analysis of the treated mice indicated they were anemic in addition to neutropenic. We feel this could confound our interpretation since we now believe FumC’s importance lies in its ability to function under iron-limitation, however we do not have evidence to fully support this so we have revised the manuscript in line 447 to say “While blood tests indicated that the pharmacological depletion of neutrophils was successful and we observed a 3-4 log increase in total CFUs comparing cyclophosphamide- to PBS-treated mice (Fig. S6A and C), the observed fitness defects in *sdhB* and *fumC* were not reversed by neutrophil depletion (Fig. S6B and D).” As indicated these new data are included as the new Figure S6.**

 *Reviewer #2: 1. The title refers ‘flexible metabolism and respiratory pathways’ as key contributors to E. coli fitness during UTI. In the end, the two gene products that are the focus of much of the study, FumC and SdhB, are associated with oxidative stress or changes in membrane integrity. More direct evidence to back up these possibilities (i.e. using genetic reporters or chemical sensors) would greatly strengthen the paper and distinguish it from the authors previous work implicating the TCA cycle as a fitness determinant during UTI.*

**I’m afraid to say that we agree with this point. We have made (in our view) substantial efforts to clean up this study and to provide additional evidence to support our hypotheses. We have now specifically provided key evidence that perhaps the TCA cycle is not as clearly essential as previously touted since loss of SDH and FRD together are equally fit with the parental strain. FRD is actually conditionally detrimental in the absence of SDH, rather than SDH being important in and of itself. The revised study is now more sharply focused on FumC and the revised title now reflects this instead of the aforementioned “flexible metabolism and respiratory pathways” that admittedly lacked clarity. The new title is “The Oxidative Fumarase FumC is a Key Contributor for *E. coli* Fitness under Iron-limitation and during UTI”.** *2. The authors suggest that the altered sensitivities of some of the mutant strains to antibiotics is associated with changes in respiration or altered electrochemical gradients across the bacterial membrane (Fig 10 and S6). Do the mutants display differential sensitivity to antibiotics under anaerobic conditions?*

**That is a very good question. The answer is yes. Interestingly, the differential sensitivity was very consistent between aerobic and anaerobic conditions. We observed a general trend toward reduced susceptibility to bactericidal antibiotics across all of the strains tested (Fig. S8). As expected, we observed a dramatic increase in resistance to the aminoglycoside streptomycin under anaerobic conditions where the parental CFT073 MBC shifted over 10-fold from 4 µg/ml to 48 µg/ml (Fig. 10F and S8C). This indicates that the decreased PMF that occurs during anaerobic growth likely results in decreased uptake of the aminoglycoside and reduced susceptibility, which was expected because it is known that aminoglycosides are ineffective toward anaerobic microbes. However, similar to what was observed under aerobic conditions, any strain lacking FumC was more resistant to streptomycin than the parental strain; *fumC* 79 µg/ml, *fumAC* 128 µg/ml, *fumBC* 112 µg/ml, and *fumABC* 128 µg/ml (Fig. S8C). The results for ampicillin and ciprofloxacin under anaerobiosis were also similar to what was observed under aerobic conditions, where double and triple mutants containing a disruption in *fumC* were 2- or 3-fold more resistant than the parental strain (Fig. 10D and E, Fig. S8A and B). While far from conclusive, the collective results from the MBC experiments demonstrate some unappreciated linkage between the TCA cycle and the mechanism underlying susceptibility to these bactericidal antibiotics that may be exclusive of reactive oxygen. This point is also discussed in line 684-702 in the revised manuscript. These new data are shown below and are in the new Fig. S8 as indicated.**

*3. The Fim switch is nearly entirely OFF in the sdhR and fumC mutants (Fig 11C), yet fimA transcription is for the most part unaffected in these mutants (Fig S8E). The reason for this dis-connect is not clear. Please clarify.*

**Review #2 brings up a valid point about the data which can be explained. The invertible element data collected in Figure 11C (and Supplement 9A) was obtained by static cultures in stationary phase, which promote the expression of Type 1 fimbriae. The gene expression data in figure S10E was obtained during mid-log phase in shaking culture, in which Type 1 expression is known to be suppressed. Therefore, since we normalized the data in each experiment relative to wild-type in each given condition, we would expect the differences to be less pronounced in the mid-log condition.**

**It is notable that the data in Figure S10E is presented in Log_2_ fold-change, the *sdhB* mutant is only displaying 34% expression of *fimA* relative to the wild-type and 12% for *fumC*.**

***Part III – Minor Issues: Editorial and Data Presentation Modifications*** *Please use this section for editorial suggestions as well as relatively minor modifications of existing data that would enhance clarity.

Reviewer #1: 4. The use of the term “fumarate dehydratase” is a bit unusual (there is no water removed from the fumarate molecule). This enzyme is typically called fumarase or fumarate hydratase as it (inter)converts fumarate plus water to malate.*

**Quite right and how embarrassing that we were careless with this in the manuscript. Throughout the revised manuscript, all three enzymes are now referred to as fumarase to improve the clarity for readers. We thank reviewer #1 for making this point. We have replaced the term “fumarate dehydratase” as a designation for the Fum enzymes to fumarase.**

*5. Do lystates of the fumABC mutant lack fumarase activity? It is conceivable that other, uncharacterized genes might encode an alternative fumarase or that other hydratases exhibit moonlighting activity.*

**Another great point, we obviously have not seriously considered moonlighting enzymes. When searching the thoroughly annotated CFT073 genome, the enzymes most similar to fumarase are AspA (c5222) and PurB (c1510). Aspartate ammonia-lyase (AspA) catalyses the conversion of L-aspartate to fumarate +NH_3_ and adenylosuccinate lyase (purB) also generates fumarate as product. While both of these enzymes may be contributing to the pool of intracellular fumarase, neither has the ability to convert fumarate to L-malate. We must also acknowledge that we have not yet explored the glyoxylate shunt, which could be important during growth on acetate or similar carbon sources. However, we do discuss some these limitations in our study in line 712 of the discussion.**

**Since most all of our phenotypes can be explained by the presence or absence of fumC, we feel it may not be necessary to explore the enzymatic capacity of the triple mutant for this present study. It is notable that we can complement the triple mutant *in vitro* by the re-introduction of *fumC* in trans. Further, the lack of growth of the triple mutant in MOPS with glucose suggests that if there is a moonlighting function it is not evident in that specific condition.**

*6. Fig. 1E and other figures: It is hard to see which symbols are open or closed.*

**Thank you. All animal data (Figure 2 & 3) have been adjusted to display kidney colonization as open squares and bladder data are closed circles to make this more distinguishable. Additionally, the symbol sizes in Fig. 2 & 3 have been enlarged. This is also addressed in other figures, outlined below in Reviewer #2 minor point #4.**

*Reviewer #2: 1. The paper could probably be shortened substantially to improve readability and clarity. As is, it is difficult to work through the mountain of data and text. Discussion is too much of a recap of the Results section and could be shortened.*

**Upon re-reading the manuscript in its entirety, we strongly agree with Reviewer #2 (we are very grateful for the patience of this reviewer and reviewer #1). We have removed roughly 550 words from the discussion with a focus on eliminating redundancy with statements were made in the results section. We believe this makes the discussion more clear and easy for readers to follow. We have also greatly emphasized FumC, which we believe also will improve the experience for potential readers.** *2. Many of the reported phenotypes are modest (small effect sizes), yet the authors use words like “crippled” when referring to slightly attenuated mutant strains. This should be adjusted.*

**We agree with Reviewer #2, the term crippled was a big overreach on our part. To address this, the word crippled has been changed in line 259, 274, 512, and deleted from line 655 of the original submission. We kept line 300 (current manuscript) because we felt that this use was an appropriate descriptor for the extreme growth defect observed for the triple mutant in Figure 6B.**

*3. The fumC mutant lags behind the wild-type strain, but reaches the same OD as the wild-type strain (Fig 6) regardless of the addition of iron. On page 14, the text suggests that this is only seen when iron is added (lines 284-286). This is not the case, based on the data in Fig 6.*

**Fair point. We agree completely and have revised this to read in line 290: “With addition of iron in the medium the growth of each strain was improved compared to growth in iron-free conditions, however; the *fumC* mutant still possessed a lag in growth during iron conditions yet was able to reach a final OD saturation similar to wild-type (fig. 6A).”** *4. Some of the symbols/lines in some of the graphs are difficult to discern (e.g. Fig. 10B). These could be better distinguished to help the reader.*

**Figures 10 A, B and C, Figure S7 A, B, C and Figure 9A have been altered to allow for better readability and display larger symbol sizes for clarity. Please also see response to Reviewer #1 minor point #6.**

*5. Why is 25C used for the phenol red agar plate assays?*

**Good question. We initially incubated aerobically at 37^o^C, however we noticed that the end products began to diffuse further into the agar away from the colonies making it difficult to discern any differences. We therefore adjusted the protocol and used room temperature incubation to limit the size of the colonies and therefore reduce the diffusion problem. We still encountered minor issues with diffusion as seen in Figure 7B panel with 48h data.**

*6. This study and the previous two studies by the Mobely group on TCA-associated enzymes use a single UPEC isolate, CFT073. The impact and generality of this study would be elevated if some of the key findings were corroborated in one or more additional strains, such as an ST131 isolate.*

**That is a very fair point. While we don’t disagree, we do feel it is a little beyond the scope of this study. Since we are studying highly conserved pathways and enzymes, we feel the ability to generalize our findings is appropriate. If this were a study of a virulence factor such as a fimbrial type or iron acquisition with known or unknown prevalence across isolates, then we believe that would be a greater concern. Indeed, one of our previous studies showed that *fumC* was also important for fitness during UTI of another far more distant relative, *Proteus mirabilis*.**
